# French Version of the User Mobile Application Rating Scale: Adaptation and Validation Study

**DOI:** 10.2196/63776

**Published:** 2024-10-24

**Authors:** Ina Saliasi, Romain Lan, Maryem Rhanoui, Laurie Fraticelli, Stéphane Viennot, Delphine Tardivo, Céline Clément, Benjamin du Sartz de Vigneulles, Sandie Bernard, Adeline Darlington-Bernard, Claude Dussart, Denis Bourgeois, Florence Carrouel

**Affiliations:** 1 Laboratory Health Systemic Process (P2S), UR4129 Research Unit, University Claude Bernard Lyon 1, University of Lyon, Lyon, France; 2 Anthropologie Bio-Culturelle, Droit, Éthique et Santé Laboratory (ADES, UMR7268), Aix Marseille University, Centre National de la Recherche Scientifique, Etablissement Français du Sang, Marseille, France; 3 Hospices Civils of Lyon, Lyon, France; 4 Laboratory “Interpsy”, UR4432, University of Lorraine, Nancy, France

**Keywords:** mHealth, mobile health, mobile health apps, eHealth, Mobile Application Rating Scale, user version, mobile apps, quality assessment tool, uMARS

## Abstract

**Background:**

Managing noncommunicable diseases effectively requires continuous coordination and monitoring, often facilitated by eHealth technologies like mobile health (mHealth) apps. The end-user version of the Mobile Application Rating Scale is a valuable tool for assessing the quality of mHealth apps from the user perspective. However, the absence of a French version restricts its use in French-speaking countries, where the evaluation and regulation of mHealth apps are still lacking, despite the increasing number of apps and their strong relevance in health care.

**Objective:**

This study aims to translate and culturally adapt a French version of the user Mobile Application Rating Scale (uMARS-F) and to test its overall and internal reliability.

**Methods:**

Cross-cultural adaptation and translation followed the universalist approach. The uMARS-F was evaluated as part through a cohort study using the French mHealth app “MonSherpa” (Qare). Participants were French-speaking adults with Apple or Android phones, excluding those with difficulty understanding French, prior app use, or physical limitations. They assessed the app using the uMARS-F twice (T1 and T2) 1 week apart. Scores for each section and overall were assessed for normal distribution using the Shapiro-Wilk test and presented as mean (SD), and potential floor or ceiling effects were calculated accordingly. Overall reliability was evaluated using intraclass correlation coefficients and internal reliability using Cronbach α. Concordance between the 3 subscales (objective quality, subjective quality, and perceived impact), 4 sections, and 26 items at T1 and T2 was evaluated using the paired *t* test (2-tailed) and Pearson correlation.

**Results:**

In total, 167 participants assessed the app at both T1 and T2 (100% compliance). Among them, 49.7% (n=83) were female, and 50.3% (n=84) were male, with a mean age of 43 (SD 16) years. The uMARS-F intraclass correlation coefficients were excellent for objective quality (0.959), excellent for subjective quality (0.993), and moderate for perceived impact (0.624). Cronbach α was good for objective quality (0.881), acceptable for subjective quality (0.701), and excellent for perceived impact (0.936). The paired *t* tests (2-tailed) demonstrated similar scores between the 2 assessments (*P*>.05), and the Pearson correlation coefficient indicated high consistency in each subscale, section, and item (*r*>0.76 and *P*<.001). The reliability and validity of the measures were similar to those found in the original English version as well as in the Spanish, Japanese, Italian, Greek, and Turkish versions that have already been translated and validated.

**Conclusions:**

The uMARS-F is a valid tool for end users to assess the quality of mHealth apps in French-speaking countries. The uMARS-F used in combination with the French version of the Mobile Application Rating Scale could enable health care professionals and public health authorities to identify reliable, high-quality, and valid apps for patients and should be part of French health care education programs.

## Introduction

Noncommunicable diseases (NCDs), such as cardiovascular diseases, diabetes, cancers, and chronic respiratory conditions, represent a major burden for global health systems and account for 74% of all deaths according to the World Health Organization [1]. The management of NCDs is challenging, requiring complex coordination among various medical stakeholders and regular monitoring of treatments and symptoms outside of hospitalization periods [2].

In the context of new global health care challenges such as demographic changes due to an aging population and a shortage of health care professionals (HCPs), particularly in rural areas, the need to implement out-of-hospital assessment and health monitoring systems to ensure continuous and effective communication between patients and HCPs is critical [3]. eHealth technologies, especially mobile health (mHealth) apps, offer promising solutions to address this need [4,5] and can be applied not only to NCDs but also to health determinants (eg, sleep, diet, exercise, and mental health).

In secondary and tertiary prevention, mHealth apps are revolutionizing the way clinicians and researchers monitor and manage the risk of recurrence or complication [6-9], reducing health inequalities by facilitating access to quality health care [10], improving lifestyle behaviors and chronic condition management, lowering health care costs [11], and increasing patient awareness and autonomy [12,13]. In primary prevention, individuals empower themselves to achieve better health conditions. Despite the growing popularity of mHealth apps and their promising outlook, most of them are unregulated and have not been scientifically evaluated in terms of effectiveness, efficiency, cost, and patient acceptability [14]. The 2016 best practice guidelines by the French National Authority for Health (Haute Autorité de Santé) were an important step toward improving the quality of mHealth apps [15]. These guidelines aimed to provide developers with a framework to ensure apps meet certain standards in terms of functionality, data privacy, and safety but without a clinical purpose. However, the guidelines are nonbinding and do not constitute formal regulation [16]. This means that adherence is voluntary, and there are no legal mechanisms to enforce compliance or monitor apps after launch, which creates gaps in quality assurance. Moreover, while the guidelines outline broad principles, they lack specific evaluation criteria or a standardized certification process, making it difficult for health care providers and users to assess whether an app meets the recommended standards. This creates variability in app quality and can lead to the promotion of apps that may not have been rigorously tested or validated. The absence of mandatory oversight means that some apps might pose risks to users, particularly regarding data security and clinical accuracy.

On the other hand, this lack of evaluation significantly impacts on the difficulties encountered by clinicians and end users face in selecting safe and effective apps [3]. The quality of the information is questionable, and the developer’s evaluation is not comprehensive enough to help end users, HCPs, and researchers identify the app’s quality [17,18].

Publicly available information, star ratings that may be artificially inflated, or downloads are the most common ways to select a mHealth app rather than validated scientific content [19]. Only a few mHealth apps available on the market have undergone a thorough validation process based on high-level evidence [20]. Appropriate quality and efficacy assessment and assurance are therefore needed both during the development and ongoing use of mHealth apps [21].

To objectively evaluate the validity and functionality of mHealth apps, several standardized scales have been developed for HCPs [22]. One of the most widely used of these assessment tools, the Mobile Application Rating Scale, developed by Stoyanov et al [22], is to date considered as the reference scale for HCPs in the scientific literature. However, this scale requires a level of scientific and clinical expertise and training in mHealth, making it almost impossible for its use by end users. Therefore, the same team of authors developed the end-user version of the Mobile Application Rating Scale (uMARS), which is a valid and objective tool that can be used by end users with different levels of education or by researchers working with end users to evaluate and assess the quality of mHealth apps from end-user perspective [23]. This scale is available in English (uMARS) [23], Spanish (uMARS-S) [24], Italian (uMARS-I) [25], Japanese (uMARS-J) [26], Turkish (uMARS-T) [27], and Greek (uMARS-G) [28]. The cross-cultural translation and validation of the original uMARS into the French language have not yet been carried out, limiting its use in French-speaking countries, despite the fact that 321 millions of people worldwide speak French, representing the fifth most spoken language in the world [29]. The aim of this study was (1) to translate and culturally adapt a French version of the uMARS and (2) to test its overall and internal reliability.

## Methods

### Study Design

This study followed and applied the universalist approach [30] to translate, cross-culturally adapt, and validate the original version of uMARS [23] into French. The reliability of the French version of the user Mobile Application Rating Scale (uMARS-F) was assessed through a prospective, longitudinal cohort study, adhering to the STROBE (Strengthening the Reporting of Observational Studies in Epidemiology) guidelines (Multimedia Appendix 1).

### Description of the uMARS

The uMARS is a valid and useful tool designed to allow end users to evaluate the quality of English-language mHealth apps [23]. It consists of 26 items organized into 3 subscales. The first one, the subscale (“objective quality”) is divided into 4 sections: engagement (items 1-5), functionality (items 6-9), aesthetics (items 10-12), and information (items 13-16). The second one, the subjective subscale (“subjective quality”) contains 4 items (17-20). The third one, an additional 6-item subscale measuring the app’s perceived impact on awareness, knowledge, attitudes, intention to change, help-seeking, and probability of changing the targeted health behavior. Each item of the uMARS is rated on a 5-point Likert scale ranging from 1=poor to 5=excellent. The response “not applicable” (NA) is an additional option included for the information section in case an item may not be applicable. The uMARS scale is both a profile, as each subcategory has a score, and an index, as it provides a final overall score for assessing the quality of mHealth care apps.

### Process of Translation and Cultural Adaptation

**Figure 1.**
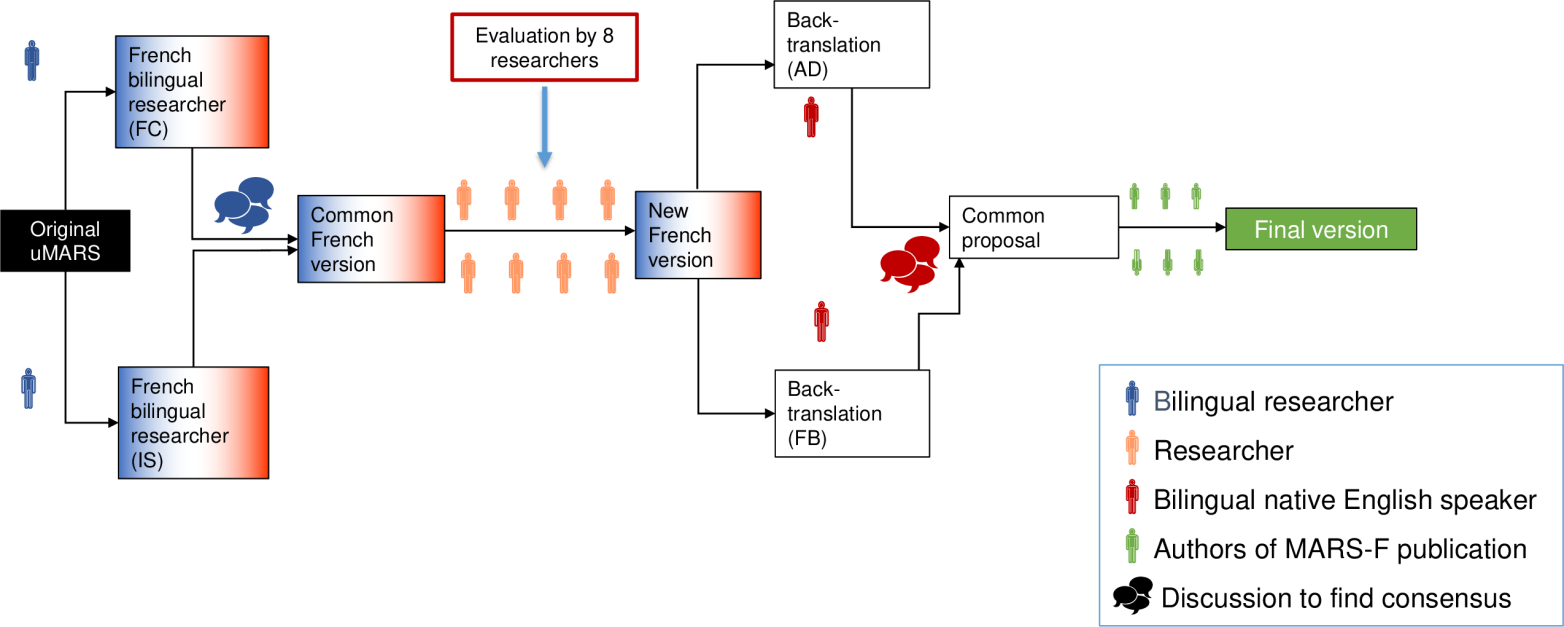
Translation methodology for uMARS. MARS-F: French version of the Mobile Application Rating Scale; uMARS: user version of the Mobile Application Rating Scale.

Figure 1 presents the process of translation and cultural adaptation of the uMARS in the French language. Two native French bilingual researchers (FC and IS) independently translated the original version of the uMARS from English into French. The 2 French translations obtained were compared by the 2 researchers who, in case of discrepancies, discussed to ensure that the meanings were as close as possible to the original English version and proposed a common French version after reaching a consensus. The common version’s comprehensibility was then evaluated by 8 researchers (DB, LF, CC, SB, DT, SV, BDSDV, and CD) who, after discussion, agreed on a new consensual French version. This version was blind back-translated in English by 2 native English speakers (AD-B and Felicor Bongolan) independently. Their final common proposal was reviewed and compared with the original English version, which was then read and evaluated by 6 researchers, authors of the French version of the Mobile Application Rating Scale (MARS-F; IS, FC, LF, DB, CD, and DT). Their comments and suggestions were discussed within the research group, and the final version of the uMARS-F was developed.

### Evaluation of Reliability

The reliability of the uMARS-F was evaluated between December 2022 and June 2023 using the French mHealth app “MonSherpa” (Qare). This app, chosen by the research team, is a free psychological support app available in the Google Play and Apple stores, targeting adults (≥18 years) of both sexes.

The sample size for validation of the uMARS-F scale was calculated to ensure sufficient statistical power to assess reliability. Using test-retest reliability with intraclass correlation coefficients (ICCs) in a 2-way random-effects, k-measurement model with consistency, the target was to detect an ICC of 0.75 with 80% power and a 5% significance level, with 2 measures per participant. Based on standard calculations, a minimum of 35 participants was required to achieve this level of reliability.

The recruitment method was based on voluntary participation. Participants were initially enrolled through the established network of research laboratory members, who received an email inviting them to participate. Additionally, social networks such as LinkedIn and Facebook were used to reach a broader audience. A snowball effect was also used, where initial participants were encouraged to refer others who might be interested.

The inclusion criteria were (1) French people aged 18 years and older, (2) persons having access to an Apple or Android phone, and (3) people who did not know the app or had not used it before. The exclusion criteria were people who (1) have difficulty in understanding the French language, (2) already know and used this app personally, and (3) had difficulty using “MonSherpa” for any reason (eg, physical disability).

Eligible participants were asked to download the “MonSherpa” app and use it at least 5 minutes a day for 1 week. On the seventh day, they rated (time 1, T1) this app using a web-based version of the uMARS-F. Then, they were not allowed to use this app for 1 week. Finally, after this washout period, all the participants used this app for only 5 minutes and rated it once again using the same scale (time 2, T2).

### Data Analysis

#### Descriptive Statistics

The distribution of summary scores for each section and for all sections was evaluated for normal distribution using the Shapiro-Wilk test. For the normal distribution, means and SDs were calculated. Floor or ceiling effects were present when more than 15% of responses were rated as the minimum or maximum scores, respectively [31].

#### Evaluation of the Validity and Reliability of the uMARS-F

##### Intraclass Correlation Coefficient

The ICCs were calculated to assess the interrater reliability of the sections and subscales. A random-effects average measures model with absolute agreement was used for this calculation. ICC values were interpreted as follows: less than 0.50 indicated poor reliability, 0.51-0.75 indicated moderate reliability, 0.76-0.89 indicated good reliability, and greater than 0.90 indicated excellent reliability.

##### Cronbach α

The internal consistency of the uMARS-F questionnaire was evaluated using Cronbach α. A high Cronbach α indicates that the items within the scale are well correlated, suggesting that the scale is reliable. The interpretation of the Cronbach α coefficient was as follows: excellent (≥0.90), good (0.80-0.89), acceptable (0.70-0.79), questionable (0.60-0.69), poor (0.50-0.59), and unacceptable (<0.50) [31].

##### Pearson Coefficient

The test-retest reliability was evaluated using Pearson *r* coefficients with 95% CIs. The correlation coefficient ranges between –1 and 1, with values closer to 1 indicating a strong positive linear relationship between the subscales, sections, and items, and values closer to –1 indicating a strong negative linear relationship. The significance of the correlations was assessed to determine the strength of the association.

##### Paired *t* Test

The paired *t* test (2-tailed) was used to compare the means of the 2 times and evaluate the test-retest reliability. This test assesses whether the mean difference between paired observations is significantly different from 0. A *P* value less than .05 indicates that there is a statistically significant difference between the paired means.

### Statistical Software

The statistical analysis was carried out using Python (version 3.10; Python Software Foundation), with scipy.stats for statistical calculations and matplotlib along with seaborn for data visualization.

### Ethical Considerations

This study did not involve research with access to health data and, therefore, falls outside the scope of the French Jarde law on research involving humans (Law 2012‐300 of March 5, 2012). Participants gave their consent by completing the web-based questionnaire, and they could withdraw from the study at any time by logging out. Data were completely anonymous, as no identifying data were collected. The platform Claroline Connect from the University Claude Bernard Lyon 1 used for the web-based questionnaire was in accordance with the Regulation EU 2016/679 of the European Parliament and the Council of April 27, 2016, on the protection of individuals with regard to the processing of personal data and on the free movement of such data. Participants received no compensation.

## Results

### Cross-Cultural Adaptation and Translation Process

Both the conceptual analysis and the translation were considered relevant and appropriate to French culture. No major differences were found between the 2 independent translations of the uMARS into French. The back-translated version of the uMARS-F was equivalent to the original uMARS except for softening changes. The final version of the uMARS-F was produced after review by the authors and mutual agreement on any discrepancies (Multimedia Appendix 2).

### Participant Characteristics

In total, 167 participants assessed the app at both time 1 and time 2 (100% compliance). Among them, 49.7% (n=83) were female, and 50.3% (n=84) were male, with a mean age of 43 (SD 16) years. In terms of education level, 21.6% (n=36) of participants did not have the baccalaureate, 25.1% (n=42) had education levels ranging from the baccalaureate to 2 years postbaccalaureate, and 53.3% (n=89) had education levels higher than 2 years postbaccalaureate.

### Descriptive Results of the uMARS-F Assessment

All the participants have filled out the uMARS-F questionnaire twice, 7 days apart. They answered all the questions. The mean uMARS-F objective quality score was 4.08 (SD 0.77), whereas the subjective quality score was 2.95 (SD 1.20), and the perceived impact score was 3.42 (SD 0.96). There were no missing values among the responses.

The descriptive analysis of the evaluation is presented in Table 1. A high ceiling effect was observed for the objective quality and its 4 sections (engagement, functionality, aesthetics, and information), suggesting that respondents tended to give higher ratings, indicating overall satisfaction. No floor effect was observed except for the subjective quality (n=26, 15.6%). The Shapiro-Wilk test showed a lack of fit to the normal distribution in subscales and sections.

**Figure 2.**
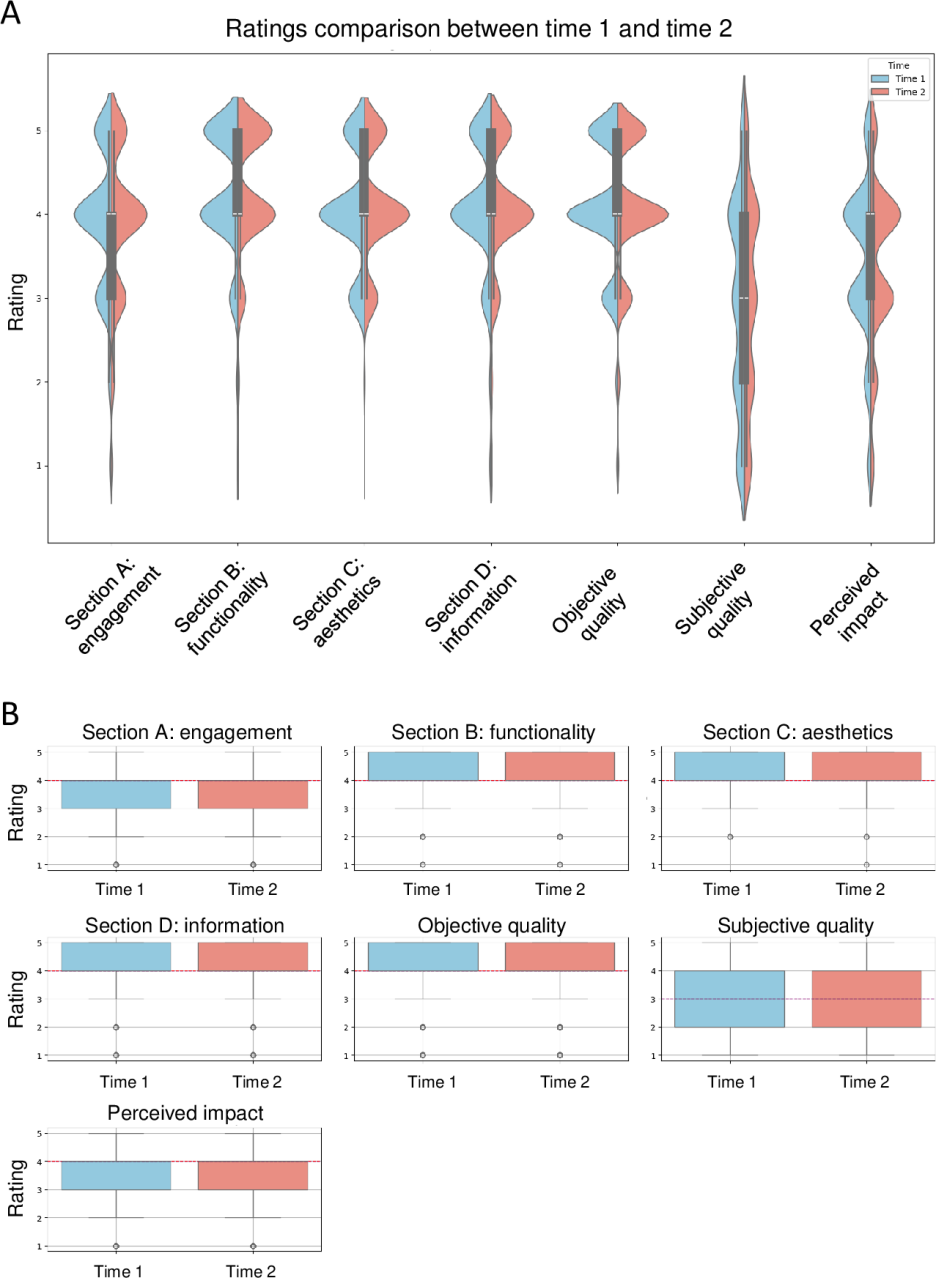
Descriptive results of the uMARS-F evaluation. (A) Violin plots of the uMARS-F subscales and section scores. (B) Box plot of the uMARS-F subscales and section scores. uMARS-F: French version of the user Mobile Application Rating Scale.

The distribution of scores across the uMARS-F subscales and sections presented in Figure 2 indicates high consistency between the 2 evaluation times (T1 and T2), as evidenced later by the strong Pearson correlations (all *P*<.001). The objective quality subscale and its sections generally exhibited scores concentrated between 3 and 5, suggesting a favorable evaluation of the app quality. However, the subjective quality subscale showed a wider range of scores from 0 to 5, indicating more variability in user perceptions. For the perceived impact section, scores were predominantly grouped between 3 and 4, reflecting a moderate perceived impact of the app.

**Table 1.** Descriptive statistics.

		Ceiling effect, n (%)	Floor effect, n (%)	Shapiro-Wilk, *P* value	Mean (SD)
**Objective quality**		50 (29.9)	2 (1.2)	<.001	4.08 (0.77)
	Section A (engagement)	38 (22.8)	3 (1.8)	<.001	3.88 (0.87)
	Section B (functionality)	70 (41.9)	1 (0.6)	<.001	4.28 (0.73)
	Section C (aesthetics)	44 (26.3)	0 (0.0)	<.001	4.07 (0.69)
	Section D (information)	47 (28.1)	3 (1.8)	<.001	4.07 (0.80)
Subjective quality		13 (7.8)	26 (15.6)	<.001	2.95 (1.20)
Perceived impact		16 (9.6)	8 (4.8)	<.001	3.42 (0.96)

### Evaluation of the Validity and Reliability of the uMARS-F

#### Evaluation of Interrater Reliability Using ICCs and Internal Consistency Using Cronbach α Coefficients

The results for the ICC and Cronbach α are described in Table 2. The ICC was excellent for the objective quality subscale (0.959, 95% CI 0.93-0.98), excellent for the subjective quality subscale (0.993, 95% CI 0.98-1.0), and moderate for the perceived impact (0.624, 95% CI 0.32-0.92). The engagement, aesthetics, and information sections obtained an excellent ICC score, whereas the functionality section obtained a moderate ICC score.

The Cronbach α was good for the objective quality subscale (0.881), acceptable for the subjective quality subscale (0.701), and excellent for the perceived impact subscale (0.936).

**Table 2.** Interrater reliability using the intraclass correlation coefficients (ICCs) and internal consistency using the Cronbach α coefficients for the French version of the user Mobile Application Rating Scale.

		ICC (95% CI)	Cronbach α (95% CI)
**Objective quality**		0.959 (0.93-0.98)	0.881 (0.862-0.899)
	Section A (engagement)	0.975 (0.93-1.0)	0.732 (0.683-0.775)
	Section B (functionality)	0.771 (0.43-0.98)	0.795 (0.757-0.829)
	Section C (aesthetics)	0.914 (0.72-1.0)	0.777 (0.732-0.816)
	Section D (information)	0.926 (0.78-0.99)	0.734 (0.684-0.777)
Subjective quality		0.993 (0.98-1.0)	0.701 (0.646-0.751)
Perceived impact		0.624 (0.32-0.92)	0.936 (0.924-0.946)

#### Paired *t* Test and Pearson Coefficient

The paired *t* test (2-tailed) and Pearson coefficient are presented in Table 3. The results from paired *t* tests (2-tailed) indicated no significant differences (*P*>.05) between T1 and T2 between the 20 items and overall subscale scores, highlighting a high test-retest reliability over time in end users’ perceptions.

**Table 3.** Paired *t* test (2-tailed) and Pearson correlation for the French version of the user Mobile Application Rating Scale.

			Paired *t* test			Pearson correlation	
			Mean score at T1 (SD)	Mean score at T2 (SD)	*P* value	*r*	*P* value
**Objective quality**			4.06 (0.80)	4.06 (0.80)	.12	0.84	<.001
	**Section A (engagement)**		3.89 (0.86)	3.87 (0.87)	.33	0.84	<.001
		1. Entertainment	3.83 (0.79)	3.86 (0.79)	.56	0.78	<.001
		2. Interest	3.89 (0.66)	3.89 (0.71)	>.99	0.76	<.001
		3. Customization	3.30 (1.01)	3.34 (0.97)	.28	0.88	<.001
		4. Interactivity	3.99 (0.76)	3.93 (0.87)	.13	0.81	<.001
		5. Target group	4.42 (0.65)	4.33 (0.73)	.05	0.79	<.001
	**Section B (functionality**)		4.27 (0.74)	4.28 (0.73)	.44	0.81	<.001
		6. Performance	4.14 (0.77)	4.16 (0.75)	.55	0.87	<.001
		7. Ease of use	4.33 (0.67)	4.31 (0.69)	.62	0.76	<.001
		8. Navigation	4.21 (0.82)	4.26 (0.81)	.18	0.84	<.001
		9. Gestural design	4.40 (0.67)	4.41 (0.64)	.87	0.74	<.001
	**Section C (aesthetics)**		4.05 (0.69)	4.09 (0.69)	.06	0.81	<.001
		10. Layout	4.22 (0.63)	4.25 (0.64)	.43	0.81	<.001
		11. Graphics	3.86 (0.71)	3.93 (0.70)	.05	0.79	<.001
		12. Visual appeal	4.07 (0.68)	4.10 (0.68)	.27	0.81	<.001
	**Section D (information**)		3.95 (0.80)	3.96 (0.77)	.81	0.89	<.001
		13. Quality of information	3.95 (0.75)	3.96 (0.73)	.67	0.88	<.001
		14. Quantity of information	3.99 (0.75)	3.97 (0.70)	.62	0.79	<.001
		15. Visual information	3.93 (0.91)	3.95 (0.89)	.41	0.95	<.001
		16. Credibility of source	3.95 (0.80)	3.96 (0.77)	.81	0.89	<.001
**Subjective quality**			2.94 (1.21)	2.96 (1.20)	.43	0.90	<.001
	17. Would you recommend		2.93 (1.06)	2.97 (1.08)	.41	0.81	<.001
	18. How many times		3.43 (1.02)	3.43 (1.00)	.89	0.85	<.001
	19. Would you pay		1.69 (0.77)	1.74 (0.81)	.15	0.82	<.001
	20. Overall (star) rating		3.72 (0.82)	3.69 (0.85)	.51	0.85	<.001

Pearson correlations between T1 and T2 are consistently high (ranging from 0.74 to 0.95), indicating strong positive relationships and high reliability of responses across both time points. These correlations are significant (*P*<.001), reinforcing the reproducibility of end users’ evaluations over time.

## Discussion

### Principal Results and Comparison With Prior Work

The widespread use of mHealth apps over the last few years has led to a significant revolution in the treatment of lifestyle-related disorders and NCDs, which are now an inevitable part of our modern society [12]. The mHealth apps are an effective health care approach, and their number increased dramatically, accentuated since the COVID-19 crisis [32]. By the end of 2023, there were over 100,000 mHealth apps on the global market, and this number is constantly growing [33]. The French mHealth sector has followed this trend with a growing number of mHealth apps and a high level of interest in their importance for health care over the last 5 years [34]. Therefore, it is crucial to have a standardized and reproductible evaluation scale to identify those that are effective and comply with medical standards [34]. These evaluations must be conducted at 2 main levels: by HCPs, who can assess the quality of the content and its alignment with medical recommendations using the MARS-F; and by end users using the uMARS-F to evaluate the usability, acceptability, and impact on their engagement in care [7,35,36].

To date, only the MARS-F scale for HCPs [21] is available in French. The aim of this study was to translate, culturally adapt, and validate the French version of the uMARS to enable end users to assess the quality of mHealth apps. For this, a methodology similar to that used for the uMARS-J [26], uMARS-S [24], uMARS-I [25], uMARS-T [27], and uMARS-G [28] scales was followed.

The number of participants completing the questionnaires at T1 and T2 (n=157) was far higher than the calculated minimum sample size (n=35). Consequently, as similar studies have included between 35 [26] and 216 participants [24], it was decided to include all participants and analyze their data to increase the power of the results.

The validation process showed high ceiling effects for objective quality and its 4 sections, with many participants giving high scores, limiting the scale’s ability to distinguish top performers. In contrast, subjective quality had a floor effect, indicating dissatisfaction with the personal appeal of the app. The ceiling or floor effects were only observed in the Japanese version [26] and demonstrated no effect.

The reliability of the measures was primarily indicated by the ICC values. The excellent ICC values for most of the sections reflected strong interrater reliability, ensuring that the repeated measures are consistent. The uMARS-F demonstrated higher ICC values for both the objective and subjective quality subscales compared to the English, Spanish, Italian, and Japanese versions [23-26]. The comparison with Turkish and Greek versions was not possible due to unavailable ICC values [27,28].

The higher ICC values observed in the French version of the uMARS compared to the other versions may stem from cultural differences in how users rate apps. Another factor could be the translation and adaptation process of the uMARS into French which can involve, or not, careful attention to linguistic nuances and cultural relevance, contributing to more consistent ratings. This is particularly relevant for the English and Spanish versions, where there are significant linguistic disparities between countries. Additionally, the demographics and characteristics of the study samples (eg, educational levels) may differ across countries, influencing how participants engage with the tool.

These differences have important implications. Higher ICC values in the French version suggest stronger internal consistency, making it a reliable tool for evaluating mHealth apps in French-speaking populations. However, it also highlights the need for localized validation efforts, as the reliability and applicability of the uMARS may vary depending on cultural and linguistic contexts. Future research could explore how these factors impact app evaluations across different regions to ensure the tool is robust and adaptable globally.

Additionally, the results highlight a significant correlation between repeated measures (T1 and T2) using paired *t* test (2-tailed) and Pearson correlation coefficients for all subscales, sections, and items. Paired *t* tests (2-tailed) were uniquely conducted in the Italian version [25] and, similar to this study, showed no statistically significant difference in each answer or group of answers between times 1 and 2 (*P*>.05). Pearson correlation coefficients were evaluated in the Italian [25] and Japanese [26] versions, showing similarly high reliability of responses across both time points (ranging from 0.74 to 0.95).

The validity of the measures is inferred from Cronbach α values, which indicates the internal consistency and the correlation between subscales. High Cronbach α values for objective quality and perceived impact suggested strong internal consistency. The internal consistency across the sections supports the validity of the measures, as it suggests that the subscales are appropriately correlated. For the objective quality subscale, the uMARS-F’s Cronbach α (0.881) was similar to that of the original uMARS and other versions, including Italian, Japanese, Spanish, Turkish, and Greek [23-28]. For the subjective quality subscale, the uMARS-F’s Cronbach α (0.701) was acceptable and comparable to the original uMARS and Japanese versions [23,26], but higher than the Spanish, Greek, and Turkish versions [24,27,28], though lower than the Italian version [25].

### Limitations

This study presents several limitations. First, only 1 mHealth app, MonSherpa, was assessed to validate the study. Further investigations are required to test or retest the uMARS-F on other mHealth apps targeting populations more representative of the general French population. Second, the uMARS-F was developed by native French speakers living in France, and its metric properties must be taken into consideration because French speakers worldwide may have different cultures with different varieties of French depending on their country, and further adaptation may be required [21].

### Conclusions

The uMARS-F is a valid tool with adequate metric properties for evaluating the quality of mHealth apps in French-speaking countries. It offers a valuable framework for both developers and researchers to assess and enhance mHealth app quality from an end-user perspective prior to market launch or after the introduction of new functionalities. Therefore, the uMARS-F serves as a cornerstone of the French mHealth field, providing opportunities to identify reliable, high-quality, and valid apps for the benefit of end users.

The integration of the uMARS-F into existing health care systems could significantly enhance mHealth app selection by HCPs and public health authorities, enabling the recommendation of high-quality mHealth apps tailored to patient needs. Moreover, incorporating the u-MARS-F into health care education programs could help future professionals make informed decisions about mHealth tools, potentially improving patient outcomes.

From a regulatory perspective, the uMARS-F could play a key role in establishing standardized quality benchmarks for mHealth apps in French-speaking regions. Developers may also use the tool to align with best practices, ensuring that apps meet both clinical and user-experience standards.

Further research should explore the use of uMARS-F alongside MARS-F in clinical and public health settings to better assess the impact of mHealth apps on patient care. Future studies could also investigate how these tools influence app development, regulation, and long-term health care integration across various French-speaking health care systems.

## Supplementary material

10.2196/63776 Multimedia Appendix 1 STROBE (Strengthening the Reporting of Observational Studies in Epidemiology) statement—checklist of items.

10.2196/63776 Multimedia Appendix 2 French version of user version of the Mobile Application Rating Scale.

## Abbreviations

HCP: health care professional
ICC: intraclass correlation coefficient
MARS-F: French version of the Mobile Application Rating Scale
mHealth: mobile health
NCD: noncommunicable disease
STROBE: Strengthening the Reporting of Observational Studies in Epidemiology
uMARS: user version of the Mobile Application Rating Scale
uMARS-F: French version of the user Mobile Application Rating Scale
uMARS-G: user version of Mobile Application Rating Scale in Greek
uMARS-I: user version of Mobile Application Rating Scale in Italian
uMARS-J: user version of Mobile Application Rating Scale in Japanese
uMARS-S: user version of Mobile Application Rating Scale in Spanish
uMARS-T: user version of Mobile Application Rating Scale in Turkish
