# Supplementary material for: French Version of the User Mobile Application Rating Scale: Adaptation and Validation Study
Source: JMIR Mhealth Uhealth. 2024 Oct 24;12:e63776. doi: 10.2196/63776 (PMC11527390; doi:10.2196/63776)
Supplement: Multimedia Appendix 2 [file mhealth-v12-e63776-s002.pdf]

Mode d'emploi :

Les évaluateurs doivent :

1. Utiliser l'app et la tester de manière approfondie pendant au moins 10 minutes ;
2. Déterminer si elle est facile à utiliser, si elle fonctionne bien et si elle fait ce qu'elle est censée faire ;
3. Examiner les paramètres de l'app, les informations du développeur, les liens externes, les fonctionnalités de sécurité, etc.

Score :

A : Engagement Score moyen = ____
B : Fonctionnalité Score moyen = ____
C : Esthétique Score moyen = ____
D : Information Score moyen = ____
* Exclure les questions notées "N/A" du calcul du score moyen.
Score moyen de la qualité de l'app ____________________ = (A + B + C + D)/4

L'échelle de *qualité subjective de l'app* peut être présentée sous forme d'éléments individuels ou d'un score moyen, en fonction des objectifs de la recherche.

Les items de l'*Impact perçu* peuvent être ajustés et utilisés pour obtenir des informations sur l'impact perçu de l'app sur les connaissances de l'utilisateur, les attitudes et les intentions de l'utilisateur concernant le comportement de santé ciblé.

Échelle d'Évaluation des Applications Mobiles : Version utilisateur (uMARS-F)

Nom de l'app mobile : ____

Entourez le chiffre qui représente le plus précisément la qualité de l'app que vous évaluez. Tous les items sont évalués sur une échelle de 5 points allant de "1.inadéquat" à "5.excellent". Sélectionnez N/A si la question n'est pas pertinente pour l'app testée.

Évaluation de la qualité de l'app mobile

SECTION A

Engagement - Amusant, intéressant, personnalisable, interactif, avec des notifications (par exemple, envoi des alertes, des messages, des rappels, des retours d'expérience, permet le partage)

1. **Divertissement : l'app est-elle amusante/divertissante à utiliser ? A-t-elle des composants qui la rendent plus amusante que d'autres apps similaires ?**

    1. Pas du tout amusante ou divertissante
    2. Plutôt ennuyeuse
    3. Ok, moyennement amusante et divertissante, permet de divertir l'utilisateur pendant un bref instant (<5 minutes)
    4. Assez amusante et divertissante, permet de divertir l'utilisateur pendant un certain temps (5 à 10 minutes au total)
    5. Très amusante et divertissante, permet de stimuler une utilisation répétée

2. **Intérêt : l'app est-elle intéressante à utiliser ? Présente-t-elle ses informations de manière intéressante par rapport à d'autres apps similaires ?**

    1. Pas du tout intéressante
    2. Pas très intéressante
    3. Ok, moyennement intéressante ; permet d'engager l'utilisateur pendant une courte durée (<5 minutes)
    4. Assez intéressante ; permet d'engager l'utilisateur pendant un certain temps (5 à 10 minutes au total)
    5. Très intéressante, permet d'engager l'utilisateur de manière répétée

3. **Personnalisation : l'app permet-elle de personnaliser les paramètres et les préférences que vous souhaitez (par exemple, le son, le contenu et les notifications) ?**

    1. N'autorise aucune personnalisation de l'interface ou nécessite la saisie d'un réglage à chaque fois
    2. Permet peu de personnalisation et limite les fonctionnalités de l'app
    3. Personnalisation de base pour un fonctionnement adéquat
    4. Permet de nombreuses options de personnalisation
    5. Permet une personnalisation complète des caractéristiques/préférences de l'utilisateur, mémorise tous les paramètres.

4. **Interactivité : l'app permet-elle à l'utilisateur de saisir des données, de fournir un retour d'information, de contenir des invitations (rappels, options de partage, notifications, etc.) ?**

    1. Aucune fonction interactive et/ou aucune réponse à la saisie de l'utilisateur
    2. Quelques fonctions interactives, mais pas assez, ce qui limite les fonctions de l'app
    3. Fonctionnalités interactives de base pour un fonctionnement adéquat
    4. Offre une variété de fonctions interactives, de retours d'information et d'options de saisie pour l'utilisateur
    5. Très haut niveau de réactivité grâce aux fonctionnalités interactives, retour d'expérience et options de saisie de l'utilisateur

5. **Groupe ciblé : le contenu de l'app (informations visuelles, langage, conception) est-il approprié au public ciblé ?**

    1. Totalement inapproprié, peu clair ou déroutant
    2. Principalement inapproprié, peu clair ou déroutant
    3. Acceptable mais pas spécifiquement conçu pour le public ciblé. Peut être inapproprié/peu clair/confus à certains moments
    4. Conçu pour le public ciblé, avec des problèmes mineurs
    5. Conçu spécifiquement pour le public ciblé, aucun problème n'a été constaté

SECTION B

Fonctionnalité - Fonctionnement de l'app, facilité d'apprendre, fluidité de navigation, logique de flux, et interface tactile de l'app

6. **Performance : Avec quelle précision/rapidité les fonctionnalités et les composants de l'app (boutons/menus) fonctionnent-ils ?**

    1. L'app ne fonctionne pas ; pas de réponse/réponse insuffisante/réponse imprécise (par exemple, interruption anormale/bugs/fonctionnalités cassées, etc.)
    2. Certaines fonctionnalités marchent, mais sont lentes ou présentent des problèmes techniques majeurs
    3. L'app fonctionne dans l'ensemble. Certains problèmes techniques doivent être corrigés/parfois lents
    4. Principalement fonctionnelle avec des problèmes mineurs/négligeables
    5. Réponse parfaite/rapide ; aucun bug technique constaté ou contient un indicateur de « temps de chargement restant » (le cas échéant)

7. **Facilité d'utilisation : dans quelle mesure est-il facile d'apprendre à utiliser l'app ; dans quelle mesure les étiquettes, les icônes des menus et les instructions sont-elles claires ?**

    1. Pas d'instruction/instructions limitées ; les étiquettes/icônes de menu sont déroutantes ; compliquées
    2. Demande beaucoup de temps ou d'efforts
    3. Demande un peu de temps ou d'effort
    4. Facile d'apprendre à utiliser l'app (ou contient des instructions claires)
    5. Capable d'utiliser l'app immédiatement ; intuitif ; simple (pas d'instructions nécessaires)

8. **Navigation : Le passage entre les écrans est-il logique ? L'app comporte-t-elle tous les liens nécessaires entre les écrans ?**

    1. Aucune connexion logique entre les écrans/la navigation est difficile
    2. Compréhensible après beaucoup de temps/d'efforts
    3. Compréhensible après un certain temps/effort
    4. Facile à comprendre/naviguer
    5. Navigation parfaitement logique, facile, claire et intuitive, et/ou dispose de raccourcis

9. **Conception gestuelle : les tapotements/balayages/pincements/défilements ont-ils un intérêt ? Sont-ils cohérents sur tous les éléments/écrans ?**

    1. Complètement incohérents/confus
    2. Souvent incohérents/confus
    3. Ok, moyennement cohérents/éléments déroutants
    4. Majoritairement cohérents/intuitifs avec des problèmes négligeables
    5. Parfaitement cohérents et intuitifs

SECTION C

Esthétique - Conception graphique, attrait visuel global, jeu de couleurs et cohérence stylistique.

10. **Mise en page : La disposition et la taille des boutons, des icônes, des menus et du contenu sur l'écran sont-elles appropriées ?**

    1. Très mauvaise conception, encombrée, certaines options impossibles à sélectionner, localiser, voir ou lire
    2. Mauvaise conception, aléatoire, peu claire, certaines options difficiles à sélectionner/localiser/voir/lire
    3. Satisfaisante, quelques problèmes de sélection/localisation/visualisation/lecture des éléments
    4. Plutôt claire, capable de sélectionner/localiser/voir/lire des éléments
    5. Professionnelle, simple, claire, ordonnée, organisée de manière logique

11. **Graphismes : quelle est la qualité/résolution des graphismes utilisés pour les boutons, icônes, menus et contenu ?**

    1. Graphismes amateurs, conception visuelle très médiocre - disproportionnée, complètement incohérente sur le plan stylistique
    2. Graphismes de faible qualité/faible résolution ; conception visuelle de mauvaise qualité - disproportionnée
    3. Graphismes et conception visuelle de qualité moyenne (style généralement cohérent)
    4. Graphismes de haute qualité/résolution et conception visuelle - principalement proportionnés, stylistiquement cohérents
    5. Graphismes et conception visuelle de très haute qualité/résolution - proportionnés, cohérents d'un point de vue stylistique

12. **Attrait visuel : quelle est l'apparence de l'app ?**

    1. Très mauvaise organisation visuelle : désagréable à regarder, mal conçue, couleurs discordantes et mal assorties.
    2. Mauvaise organisation visuelle : mal conçue, mauvaise utilisation des couleurs, visuellement ennuyeuse
    3. Ok : Organisation visuelle moyenne, ni agréable, ni désagréable à regarder
    4. Assez bonne organisation visuelle : agréable à regarder, graphisme homogène - cohérent et conçu de manière professionnelle
    5. Très bonne organisation visuelle : magnifique - très attrayante, mémorable, remarquable ; l'utilisation des couleurs met en valeur les fonctions/menus de l'app.

SECTION D

Information - Contient des informations de haute qualité (par exemple du texte, des retours d'expérience, des références) provenant d'une source crédible.

13. **Qualité des informations : le contenu de l'app est-il correct, bien rédigé et pertinent par rapport à l'objectif/au sujet de l'app ?**

    N/A Il n'y a aucune information dans l'app
    1. Non pertinent / inapproprié / incohérent / incorrect
    2. Pauvre. Très peu pertinent / approprié / cohérent / peut être incorrect
    3. Modérément pertinent / approprié / cohérent / et semble correct
    4. Pertinent / approprié / cohérent / correct
    5. Très pertinent, approprié, cohérent et correct

14. **Quantité d'information : L'information contenue dans l'app est-elle complète mais concise ?**

    N/A Il n'y a aucune information dans l'app
    1. Minimale ou très insuffisante
    2. Insuffisante
    3. Ok, mais moyennement complète ou concise
    4. Offre un large éventail d'informations, présente quelques lacunes ou des détails inutiles ; ou ne contient pas de lien vers d'autres informations et ressources
    5. Complète et concise ; contient des liens vers d'autres informations et de ressources

15. **Infographies : l'explication visuelle des concepts - à travers des tableaux/graphiques/images/vidéos, etc. - est-elle claire, logique, correcte ?**

    N/A Il n'y a pas d'infographies dans l'app (par exemple, elle ne contient que de l'audio ou du texte)
    1. Pas du tout claires/très confuses/incorrectes ou manquantes
    2. Généralement peu claires/assez confuses/incorrectes
    3. Ok, mais souvent pas claires/confuses/incorrectes
    4. Assez claires/logiques/correctes avec des problèmes négligeables
    5. Parfaitement claires/logiques/correctes

16. Crédibilité : Les informations contenues dans l'app semblent-elles provenir d'une source crédible ?

N/A Il n'y a aucune information dans l'app

1. Source suspecte
2. Manque de crédibilité
3. Non suspect mais la légitimité de la source n'est pas claire
4. Il est possible qu'il provienne d'une source légitime
5. Provient certainement d'une source légitime/spécialisée

Qualité subjective de l'app

SECTION E

17. Recommanderiez-vous cette app aux personnes qui pourraient en bénéficier ?

1. Pas du tout Je ne recommanderais cette app à personne
2. Il y a très peu de personnes à qui je recommanderais cette app
3. Peut-être Il y a plusieurs personnes à qui je la recommanderais cette app
4. Il y a de nombreuses personnes à qui je recommanderais cette app
5. Absolument Je recommanderais cette app à tout le monde

18. Combien de fois pensez-vous que vous utiliseriez cette app au cours des 12 prochains mois si elle était pertinente pour vous ?

1. Jamais
2. 1-2
3. 3-10
4. 10-50
5. >50

19. Payeriez-vous pour cette app ?

1. Définitivement non
2.
3.
4.
5. Définitivement oui

20. Quelle note globale (étoiles) attribuez-vous à l'app ?

1. ☆ L'une des pires apps que j'ai utilisées
2. ☆☆
3. ☆☆☆ Moyenne
4. ☆☆☆☆
5. ☆☆☆☆☆ L'une des meilleures apps que j'ai utilisées

Impact perçu

SECTION F

1. **Sensibilisation - Cette app m'a permis de prendre conscience de l'importance de s'intéresser aux comportements liés à la santé**

Pas du tout d'accord				Tout à fait d'accord
1	2	3	4	5
○	○	○	○	○

2. **Connaissances - Cette app a amélioré mes connaissances et ma compréhension des comportements en matière de santé**

Pas du tout d'accord				
1	2	3	4	5
○	○	○	○	○

3. **Attitudes - L'app a changé mes attitudes envers l'amélioration de ce comportement de santé**

Pas du tout d'accord				Tout à fait d'accord
1	2	3	4	5
○	○	○	○	○

4. **Intention de changer** - **L'app a augmenté mes intentions/motivation à l'égard de ce comportement de santé**

Pas du tout d'accord				Tout à fait d'accord
1	2	3	4	5
○	○	○	○	○

5. **Recherche d'aide - Cette app m'encouragerait à demander de l'aide supplémentaire pour faire face à ce comportement de santé (si j'en avais besoin)**

Pas du tout d'accord				Tout à fait d'accord
1	2	3	4	5
○	○	○	○	○

6. **Changement de comportement - L'utilisation de cette app augmentera/diminuera les comportements de santé.**

Pas du tout d'accord				Tout à fait d'accord
1	2	3	4	5
○	○	○	○	○

Autres commentaires sur l'app ?

MERCI !
